# Relationships between physical activities performed under free-living conditions and non-motor symptoms in people with Parkinson's: A systematic review and meta-analysis

**DOI:** 10.1177/02692155241272967

**Published:** 2024-08-23

**Authors:** Amanda Still, Leigh Hale, Sarfaraz Alam, Meg E. Morris, Prasath Jayakaran

**Affiliations:** 1 School of Physiotherapy, 2495 University of Otago, Dunedin, New Zealand; 2 ARCH, 2080 La Trobe University, Bundoora, Australia

**Keywords:** Parkinson's, physical activity, non-motor symptoms

## Abstract

**Background:**

Physical activities performed under free-living conditions that are unsupervised in the home or community have the potential to modulate non-motor symptoms in people with Parkinson's disease.

**Objective:**

This systematic review investigates the relationships between physical activities performed in free-living conditions and non-motor symptoms in people with Parkinson's disease: cognition, anxiety, apathy, depression, sleep disturbances, fatigue, and pain.

**Data sources:**

A database search was performed on Scopus, Web of Science, Ovid (PsycINFO), CINAHL, PubMed, and ProQuest (Health and Medicine).

**Review methods:**

Observational studies published from 2000 to 2024 that examined the relationships between physical activity and non-motor symptoms were included. The methodological quality of reports was evaluated using critical appraisal checklists appropriate to the study design. Where appropriate, a meta-analysis was conducted to combine data from the included articles.

**Results:**

A total of 14 articles met the criteria and used various tools to evaluate non-motor symptoms and physical activity. Meta-analyses showed that people with Parkinson's who are more physically active have better global cognition [*β* ranged from 0.12 to 0.28; *p *= 0.00–0.02] and less affective disorders [*β* -0.20, *p *= 0.00]. Increased physical activity levels were also associated with better sleep quality (*n* = 1) and less chronic pain (*n* = 1). The overall methodological quality of the included articles was considered high.

**Conclusion:**

Engagement in increased levels of physical activities performed under free-living conditions is associated with better cognition and less anxiety, apathy, and depression in people with Parkinson's disease.

## Introduction

The global burden of neurological conditions is significant and increasing,^1^ and as the population ages, the prevalence of Parkinson's disease is particularly fast growing.^2^ Parkinson's is often characterised by motor impairments, such as bradykinesia and postural instability, rigidity, or tremor.^3^ In more recent times, research has highlighted the recognition of non-motor symptoms which present in more than 90% of people with Parkinson's.^4,5^ Compared to healthy older adults, the prevalence and severity of these symptoms, such as cognitive impairment, fatigue, affective disorders (e.g. depression, anxiety, apathy), pain, and sleep problems, are greater among people with Parkinson's.^6,7^ Non-motor symptoms typically increase with disease progression^8,9^ and negatively affect health-related quality of life,^10,11^ daily routines, planning/socialising, and independence.^12^

While there is growing evidence from intervention studies supporting the benefits of exercise^13–16^ and physical activities, such as dance,^17^ aquatic therapy,^18^ tai chi,^19^ and Nordic walking,^20^ for improving physical domains of health, such as muscle strength, balance, and gait speed, few have focused on non-motor symptoms as the primary outcome. Additionally, such interventions have been critiqued for being highly supervised and not reflecting a sustainable frequency or intensity of activity under free-living conditions.^21^ Although supervision ensures exercise safety and provides encouragement and support for participants, upon cessation of the intervention, many participants return toward baseline activity levels without this supervision.^21–24^ In contrast, participation in unsupervised physical activities that are performed under free-living conditions, such as gardening, housework, and community exercise classes, which are part of an individual's day-to-day life,^25–27^ tends to be more sustainable throughout the lifespan.^28–30^ The potential for participation in physical activities under free-living conditions to benefit non-motor symptoms is important, particularly when pharmaceutical interventions offer limited effectiveness or unwanted side effects.^31–36^ It is also important for people with Parkinson's, who might otherwise be sedentary due to factors, such as access or resource constraints, and low self-efficacy for exercise^37–41^; yet there is a dearth of reviews to determine this relationship.

Therefore, the purpose of this systematic review was to analyse the research literature to understand whether there is a relationship between physical activities performed under free-living conditions and non-motor symptoms in people with Parkinson's: cognitive impairment, fatigue, sleep problems, affective disorders, and pain.

## Methods

### Search and identification of articles

The review has been performed and reported according to the Preferred Reporting Items for Systematic Reviews and Meta-analyses (PRISMA) guidelines.^42,43^ The protocol has been published elsewhere.^44^

The primary reviewer (AS) conducted a systematic search (first run in October 2021, subsequently updated in June 2023 and June 2024) on the following six databases: Scopus, Web of Science, Ovid (PsycINFO), CINAHL, PubMed, and ProQuest (Health and Medicine). A keyword search strategy^44^ was designed and developed in consultation with an experienced subject librarian (TF). The search strategy, filters, and limits were tailored appropriately to the database. The full search strategy for all databases can be found in Supplementary Tables S1 and S2. Titles identified in the database search were exported to the Rayyan software. After removing the duplicates, AS screened the titles and abstracts of all citations for possible inclusion. Two reviewers (AS and PJ) then independently screened the full texts to identify relevant articles. A manual search of reference lists of included articles was carried out to locate any relevant publications that may have been missed in the electronic search. A third reviewer resolved any disagreement between the reviewers if a consensus could not be reached. Articles were considered based on the inclusion and exclusion criteria below.

### Inclusion criteria

- Adults aged 18 years and above diagnosed with idiopathic Parkinson's disease or atypical parkinsonism: atypical parkinsonism was limited to multiple system atrophy, progressive supranuclear palsy, corticobasal degeneration, and dementia with Lewy bodies;
- Cross-sectional and longitudinal studies published after the year 2000 that investigated the relationship between at least one non-motor symptom domain (i.e. cognition, affective disorders, sleep, pain, and fatigue) and at least one physical activity characteristic (e.g. metabolic equivalent minutes per week, daily steps, frequency, intensity, duration, and type);
- Studies used quantitative measurement tools to assess physical activities performed under free-living conditions and each non-motor symptom domain of interest.

### Exclusion criteria

- Studies that included participants with a diagnosis of secondary parkinsonism (e.g. vascular and drug-induced parkinsonism);
- Studies that included participants with various diagnoses (e.g. Alzheimer's and idiopathic Parkinson's disease), but did not present separate statistical analysis results for the diagnoses of interest;
- Studies on animals;
- Conference publications, abstracts, editorials, commentaries, case reports, reviews, randomised controlled trials, clinical trials, theses, or qualitative study designs;
- Studies published in languages other than English;
- Studies that used a single measurement tool for multiple non-motor symptom domains, with no individual domain scores in statistical analyses.

### Data extraction and analysis

Relevant data were extracted from the included articles by the primary reviewer (AS) using predetermined data extraction tables (Supplementary Tables S3 and S4) and verified by a second reviewer (SA). The following data were extracted: study characteristics, such as authors, year published, study design, sample size, recruitment country and setting; participant characteristics, such as diagnosis, sex, disease stage, mean (SD) age of participations, disease duration, and Levodopa dosage equivalent [LEDD]; measurement tools used to evaluate non-motor symptoms of interest and physical activity measures; and relevant statistical findings. The measures of interest were correlation and regression coefficients measuring the relationship between physical activity and the five major non-motor symptoms of interest: cognition, affective disorders, sleep, pain, and fatigue. The sleep problems of interest were sleep quality, total sleep time, and excessive daytime sleepiness. The affective disorders of interest include depression, anxiety, and apathy. The pain types of interest included musculoskeletal, dystonic, radicular, central, or akathisia. Results from univariate and multivariable analyses were included if they were reported. Physical activity categories included total weekly physical activity, daily light-intensity physical activity time, daily moderate-to-vigorous physical activity time, daily steps, daily energy expenditure, and monthly physical activity days.

### Methodological quality assessment

The methodological quality of each article was assessed independently by two reviewers (AS and SA) using either the JBI critical appraisal checklist for analytical cross-sectional studies, the JBI critical appraisal checklist for case–control studies (Supplementary Figure S1),^45^ or the Critical Appraisal Skills Programme checklist for cohort studies, as appropriate (Supplementary Figure S2).^45–47^ Any reviewer disagreements were resolved by a discussion with a third reviewer (PJ). A percentage score of methodological quality was estimated for each article. The JBI scores were classified as high quality if values were ≥70%, medium quality if between 50% and 70%, and low quality if values were <50%.^48^ However, no a-priori decision was made to exclude articles based on the methodological quality scores.

### Data synthesis

Where appropriate, a meta-analysis of continuous variables was conducted to combine data from the included articles and presented visually as forest plots. Standardised regression coefficients (*β*) were used as the effect size estimate as different measurement methods, and metrics were used across the included articles.^49^ Correlation coefficients and unstandardised regression coefficients were transformed into standardised regression coefficients using the formulas and approximations presented by Nieminen et al.^49^ (Supplementary Figure S3). The meta-analysis did not include articles reporting odds ratios (OR) or relative risks (RR).

The corresponding authors of the included articles were contacted to obtain additional data where required (e.g. *β*, 95% CI(*β*), SD(X), and SD(Y)). When *β* coefficients were not reported, it was estimated using SD(X) and SD(Y)), as recommended by Nieminen et al.^49^ Where SD(X) and SD(Y) were not attainable from authors, these were imputed from another article within this review or from another review with meta-analysis, as described by Higgins et al.^50^

To ensure that all pooled *β* coefficients are interpreted with the same directionality (i.e. higher outcome scores indicate better symptoms), *β* coefficients and corresponding 95% CIs were reversed for outcomes where a higher score indicated worse symptoms.^50^

The methodological diversity (measurement tools) and statistical diversity (statistical analyses, covariates) of the included articles were evaluated collaboratively by two reviewers (AS and PJ) to determine the homogeneity for quantitative synthesis. Initially, effect size estimates from both unadjusted and adjusted models (‘combined model’) were pooled. Where data from at least two articles were available, separate statistical pooling was undertaken for adjusted models with similar predictor variables and unadjusted models. In situations where a study reported multiple effect size estimates for the same non-motor symptom domain and physical activity association, the strongest estimate from the most comprehensive non-motor symptom measure (i.e. assessed multiple symptoms within one domain) and objective physical activity measure (e.g. estimate taken from accelerometry data over subjective recall of physical activity), which had the greatest adjustment for covariates, was selected unless this selection method reduced the homogeneity between articles, in which case, homogeneity was prioritised.

A random-effects model was used to control for any unobserved heterogeneity between articles. Further, *I*^2^ statistics were used to determine the heterogeneity of the estimates and interpreted using the following criteria: <30%, not important; ≥30%, moderate; ≥60%, substantial; and ≥90%, considerable heterogeneity.^51^ The strength of the pooled effect size (*β*) was interpreted as small if values ranged between 0.10 and 0.19, medium if between 0.20 and 0.29, and large if ≥0.30.^49^ To assess the level of certainty in the pooled effect size estimates, 95% confidence intervals (CIs) and *p-*values were reported. Meta-analyses were performed using Stata/SE 16.0 software for Mac.

## Results

### Study selection

**Figure 1.**
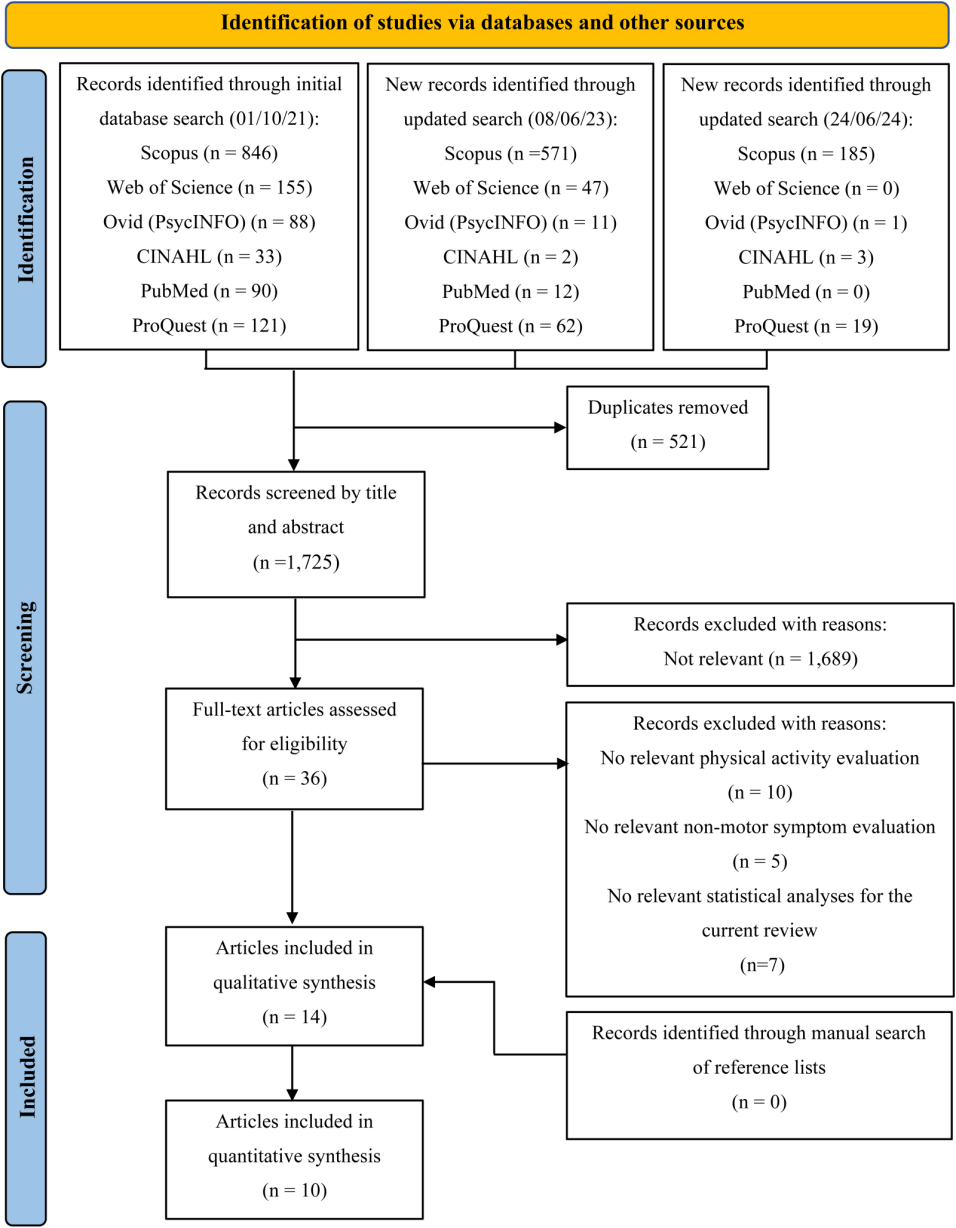
PRISMA flow diagram of the study selection process.

A total of 2246 references were identified through an electronic database search. After removing duplicates, 1752 titles were screened for relevant articles (Figure 1). The full texts of 36 articles were screened independently by two reviewers (AS and PJ) with a substantial inter-rater agreement (Cohen's *k* 0.70). After conflicts were resolved by discussion, 12 of the articles met the criteria for inclusion after the full-text screening. An additional two articles were identified from the updated searches and included in the review. The remaining 22 articles were excluded due to no relevant physical activity evaluation (*n* = 10),^52–61^ no relevant non-motor symptom evaluation (*n* = 4),^39,62–64^ or no relevant statistical analyses for the current review (*n* = 6).^39,65–71^ No additional articles were identified in the manual search of reference lists.

### Characteristics of included articles

A summary of descriptive data extracted from the 14 articles can be found in Table 1. Participants were recruited from various settings: community, local support groups, hospital-based neurology units, movement disorder clinics, Parkinson's registries, and research databases. A total of 13 investigations had participants diagnosed with idiopathic Parkinson's. One was restricted to mild Parkinson's signs.^72^ No eligible articles included individuals with atypical parkinsonism.

**Table 1.** Descriptive characteristics of included articles.

Author, year, and study design	Recruitment country	PD sample size and gender (*n*, % Male)	Diagnosis	Recruitment setting	Age, years (Mean, [SD])^a^	Disease duration, years (Mean, [SD])^a^	Disease stage, original H&Y Scale (*n,* [%])^a^	MDS-UPDRS motor examination score (Mean, [SD])^a^	LEDD, mg/day (Mean, [SD])^a^
Alwardat et al. (2019)^73^/Cross-sectional	ITA	128 (60)	IPD	Hospital-based neurology unit	61.98 (7.95)	4.99 (4.05)	NR	25.21 (10.49)^o^	493.14 (309.39)
Amara et al. (2019)^47^/Longitudinal case–control	USA, EUR	380 (65.5)	IPD	NR	63.55 (9.8)	2.6 (0.56)	0 (*n *= 2 [0.71]) 1 (*n *= 71 [25.09]) 2 (*n *= 197 [69.61]) 3–5 (*n *= 13 [4.59])	27.16 (11.3)	389.06 (302.5)
Cerff et al. (2017)^74^/Cross-sectional	DEU	PD-NC: 17 (59) PD-MCI: 22 (77) PDD: 9 (100)	IPD	Community	PD-NC: 71 (44/80)^f^ PD-MCI: 68 (57/78)^f^ PDD: 72 (67/75)^f^	PD-NC: 6 (1/13)^f^ PD-MCI: 6 (1/20)^f^ PDD: 6 (5/18)^f^	PD-NC: 1 (*n *= 6 [35]) 2 (*n *= 10 [59]) 3 (*n *= 1 [6]) PD-MCI: 1 (*n *= 3 [14]) 2 (*n *= 13 [59]) 3 (*n *= 4 [18]) 4 (*n *= 2 [9]) PDD: 2 (*n *= 3 [33]) 3 (*n *= 3 [33]) 4 (*n *= 2 [22]) 5 (*n *= 1 [11])	PD-NC: 20 (11/58)^fo^ PD-MCI: 24 (10/62)^fo^ PDD: 36 (14/ 56)^fo^	PD-NC: 620 (160/2420)^f^ PD-MCI: 763 (210/2378)^f^ PDD: 496 (100/1139)^f^
Donahue et al. (2022)^75^/Cross-sectional	USA	96 (57.3%)	IPD	NR	66.76 (8.60)	4.72 (4.81)	NR	23.99 (10.33)	568.51 (383.41)
Dontje et al. (2013)^76^/Cross-sectional	NLD	467 (66)	IPD	Hospital-based neurology unit	65.7 (7.4)	5.15 (4.38)	1 (*n *= 8 [2])^m^ 1.5 (*n *= 14 [3])^m^ 2 (*n *= 350 [75])^m^ 2.5 (*n *= 69 [15])^m^ 3 (*n *= 26 [6])^m^	32.9 (10.5)	480.0 (389.7)
Duvdevani et al. (2024)^77^/Cross-sectional	ISR	88 (65.9)	IPD	Movement Disorder Institute, Neurology Department, Health Care Campus	66.84 (8.8)	NR	1 (*n *= 4 [4.6]) 2 (*n *= 52 [59.8]) 3 (*n *= 18 [20.7]) 4 (*n *= 13 [14.9]) 5 (*n *= 0 [0])	31 (1–94)^f^	763.86 (573.01)
Ellingson et al. (2017)^78,79^/Cross-sectional	USA	52 (56)	IPD	Community	67.8 (7.9)	10.0 (6.7)	NR	57.4 (19.4)	NR
Leavy et al. (2021)^80^/Cross-sectional	SWE	89 (54)	IPD	Community and research database	71.0 (6.0)	6.0 (4.3)	NR	NR	580 (291)
Loprinzi et al. (2018)^81^/Cross-sectional	USA	23 (57)	IPD	Community	68.7 (NR)	NR	2.2 (NR)^b^	NR	NR
Ng et al. (2021)^82^/Longitudinal cohort	Singapore	121 (61.2)	IPD	Movement disorder clinics	64.5 (8.2)	NR	NR	22.33 (8.71)	186.96 (143.42)
Nguy et al. (2020)^83^/Cross-sectional	AUS	52 (69)	IPD	Community, research clinic and database	67.8 (7.8)	7.8 (6.5)	NR	33.7 (12.8)	NR
Oguh et al. (2014)^84^/Longitudinal cohort	NLD, N. Amer, IL	4866 (63)	IPD	Research database	67.0 (9.8)	5.5 (2.0–10.0)^c^	≥3 (*n *= 1699 [37.4]) ^m^	NR	NR
Santos et al. (2018)^72^/Longitudinal cohort	USA	130 (49)	MPS	Community	78.45 (7.06)	NR	NR	NR	NR
Shih et al. (2019)^85^/Cross-sectional	USA	174 (68)	IPD	Community	65.79 (9.48)	5.59 (0.54)	1.96 (0.39)^b^	29.94 (11.13)	NR

Abbreviations: *n*, number; SD, standard deviation; NR, not reported; PD, Parkinson's disease; H&Y, Hoehn and Yahr Scale; MDS-UPDRS, International Parkinson and Movement Disorder Society — Unified Parkinson's Disease Rating Scale; LEDD, Levodopa equivalent daily dosage; IPD, Idiopathic Parkinson's disease; MPS, Mild Parkinsonian Signs; PD-NC, Parkinson's disease — non-cognitively impaired; PD-MCI, Parkinson's disease — mild cognitive impairment; PDD, Parkinson's disease dementia; ITA, Italy; USA, United States of America; EUR, Europe; DNK, Denmark; DEU, Germany; NLD, Netherlands; ISR, Israel; SWE, Sweden; AUS = Australia; N. Amer, North America; IL, Israel.

a Except where indicated; ^b^Mean (SD); ^c^Mean (range); ^d^*n*,(%); ^e^Median (IQR), ^f^Median (min/max); ^m^Modified Hoehn and Yahr Scale with stages 1–5; ^o^Original UPDRS motor examination.

The sample sizes in the included studies ranged from 23 to 4866 people. All studies included females and males with a mean age that ranged from 62 to 78 years. Disease stage ranged from 0 (no clinical signs present) to 5 (wheelchair-bound or bedridden unless aided) on the original Hoehn and Yahr (H&Y) scale. The mean MDS-UPDRS part III motor score (0–132) reported by eight articles ranged from 22 to 57. The mean Levodopa equivalent daily dose reported by five articles ranged from 187 to 764 mg/day. Six studies were conducted in Europe,^47,73,74,76,80,84^ seven in North America,^47,72,75,78,81,84,85^ three in Asia,^77,82,84^ and one in Oceania.^83^

Physical activity was assessed objectively using accelerometry in seven articles^74–76,78,80,81,83^ and assessed using participant self-reported questionnaires in nine articles.^47,72,73,77,78,82–85^ The physical activity measures of interest were total weekly physical activity, moderate-to-vigorous intensity physical activity, and light-intensity physical activity.^86^ The following physical activity data were also extracted as potentially relevant: total daily steps, daily energy expenditure, and the number of days per month considered physically active.

Non-motor symptoms were typically evaluated using participant self-reported questionnaires, except for cognition, which was often evaluated using researcher-administered measures in eight articles.^47,72,74–76,81,82,85^ Relevant correlation estimates were presented in eight articles.^74–78,80,84,85^ Regression estimates were also presented in eight articles. Four of these articles^72,81–83^ reported standardised *β*-values, while the remaining four^47,73,75,84^ reported unstandardised *b*-values. The standard error (SE) of correlation (*r*_s_) and regression estimates (*β*, *b*) were not reported in any of the articles. SE (*β*) for these articles were obtained from the respective association's 95% CI. The computed standardised regression coefficient (*β*) with standard error SE (*β*) and 95% CI for each article can be found in Supplementary Table S5. Where multivariable regression models were used, estimates were usually adjusted for age, gender, Levodopa equivalent daily dose, MDS-UPDRS part III motor score, and disease duration.

The results from individual articles (Supplementary Table S5) found that increases in physical activity levels that are performed under free-living conditions were associated with better cognitive function (eight out of 11 articles),^47,72,75–77,81,84,85^ less affective disorders (five out of eight articles),^47,73,77,82,84^ better sleep quality (one out of two articles),^47^ and less pain (one out of three articles).^84^ Another article^83^ found that pain was significantly exacerbated when physical activity increased. None of the articles found a significant relationship between physical activity and fatigue or excessive daytime sleepiness.

### Combined results

**Figure 2.**
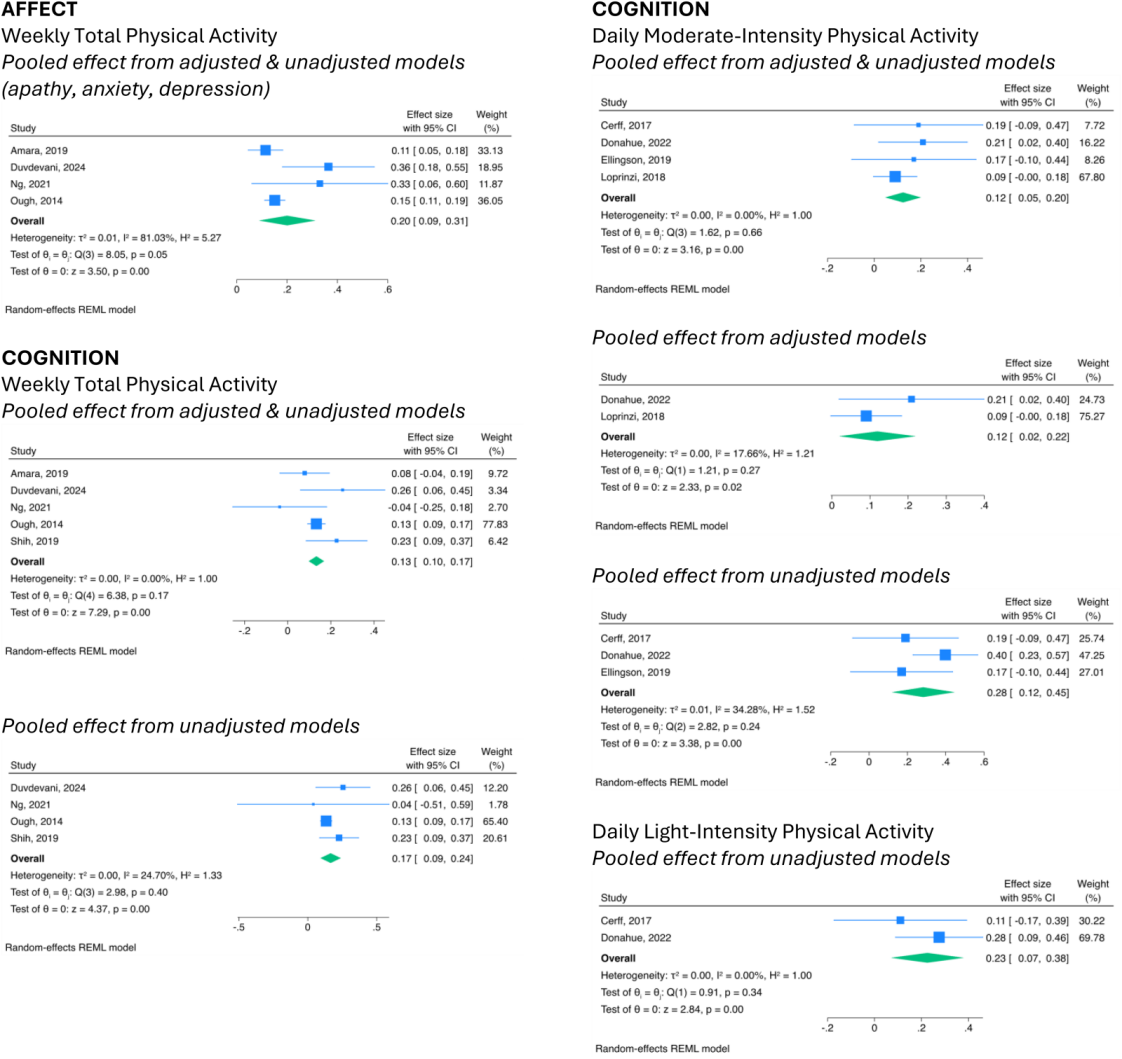
Forest plots with pooled estimates from articles estimating the relationship between physical activity categories and non-motor symptoms in Parkinson's disease.

The meta-analysis of five articles,^47,77,82,84,85^ which evaluated cognitive function, indicated significant positive effects with total weekly physical activity (combined model = *β*, 0.13; 95% CI, 0.10–0.17; *I*^2^, 0%; unadjusted model = *β*, 0.17; 95% CI, 0.09–0.24; *I*^2^, 24.70%). A significant positive relationship was also found with daily moderate-to-vigorous intensity physical activity^74,75,78,81^ (combined model = *β*, 0.12; 95% CI, 0.05–0.20; *I*^2^, 0%; adjusted model = *β*, 0.12; 95% CI, 0.02–0.22; *I*^2^, 17.66%.; unadjusted model = *β*, 0.28; 95% CI, 0.12–0.45; *I*^2^, 34.28%) and light-intensity physical activity^74,75^ (unadjusted model = *β*, 0.23; 95% CI, 0.07–0.38; *I*^2^, 0%) (Figure 2).

The meta-analysis for four articles,^47,77,82,84^ which evaluated anxiety, depression or apathy, also indicated significant positive effects with the total weekly physical activity (combined model = *β*, 0.20; 95% CI, 0.09–0.31; *I*^2^, 81.03%) (Figure 2).

No significant associations were found from the meta-analyses of articles that evaluated sleep quality (adjusted model, *p *= 0.15),^47,82^ excessive daytime sleepiness (adjusted model, *p *= 0.69),^47,82^ fatigue (adjusted model, *p *= 0.58),^47,82^ or pain (combine model, *p *= 0.78; unadjusted model, *p *= 0.63)^78,83,84^ with total weekly physical activity levels. No significant pooled effect was found between pain and moderate-to-vigorous intensity physical activity (unadjusted model, *p *= 0.49).^78,82^ See Supplementary Figure S4.

### Methodological quality assessment

All articles were classified as having high methodological quality (72.7–100%).^48^ Tables 2, 3, and 4 present the results of the methodological quality assessment for the included articles.

**Table 2.** Methodological quality assessment using the JBI critical appraisal checklist for cross-sectional studies.

Report citation	Inclusion criteria defined	Participants and setting described	Describes the use of valid and reliable exposure (physical activity) measure	PD diagnostic criteria used	Confounding factors identified	Strategies used to deal with confounding factors	Describes the use of valid and reliable outcome (non-motor symptom) measures	Appropriate statistical analyses used	Total score
Alwardat et al. (2019)^73^	Y	Y	N	Y	Y	Y	Y	Y	87.5%*
Cerff et al. (2017)^74^	Y	Y	Y	Y	Y	Y	Y	Y	100%*
Donahue et al. (2022)^75^	Y	Y	Y	Y	Y	Y	Y	Y	100%*
Dontje et al. (2013)^76^	Y	Y	Y	Y	Y	Y	Y	Y	100%*
Ellingson et al. (2017)^78^	Y	Y	Y	Y	Y	U	Y	Y	87.5%*
Leavy et al. (2021)^80^	Y	Y	Y	U	Y	Y	Y	Y	87.5%*
Loprinzi et al. (2018)^81^	N	Y	Y	Y	Y	Y	Y	Y	100%*
Nguy et al. (2020)^83^	Y	Y	Y	Y	Y	Y	Y	Y	100%*
Shih et al. (2019)^85^	Y	Y	N	Y	Y	Y	Y	Y	87.5%*

Abbreviations: Y = yes; N = no; U = unclear. Identification of confounding factors was rated ‘Y’ if typical confounders, such as age, gender, Levodopa dosage equivalent, disease duration, and motor symptom severity, were identified and measured. The use of valid and reliable measures was rated ‘Y’ if psychometric properties have been previously evaluated and shown acceptable levels in people with Parkinson's disease. Study quality is defined as follows: a total score greater than 70% as high quality, a score between 50% and 70% as medium quality, and a score less than 50% as low quality. *High quality articles, >70%.

**Table 3.** Methodological quality assessment using the JBI critical appraisal checklist for case–control studies.

Report Citation	Addressed a clearly focused issue	Acceptable recruitment	Exposure (physical activity) accurately measured	Outcomes (non-motor symptoms) accurately measured	Important confounding factors identified	Strategies used to deal with confounding factors	Was the follow-up of subjects complete enough	Was the follow-up of subjects long enough	Do you believe the results	Can the results be applied to the local population	Results fit with other available evidence	Total score
Ng et al. (2021)^82^	Y	Y	N	Y	Y	Y	Y	Y	Y	Y	Y	90%
Oguh et al. (2014)^84^	Y	Y	N	Y	Y	Y	U	Y	Y	Y	Y	81.2%
Santos et al. (2018)^72^	Y	N	N	Y	Y	Y	N	Y	Y	Y	Y	72.7%

Abbreviations: Y = yes; N = no; U = unclear. Identification of confounding factors was rated ‘Y’ if typical confounders, such as age, gender, Levodopa dosage equivalent, disease duration, and motor symptom severity, were identified and measured. The use of valid and reliable measures was rated ‘Y’ if psychometric properties have been previously evaluated and shown acceptable levels in people with Parkinson's disease. Study quality is defined as follows: a total score greater than 70% as high quality, a score between 50% and 70% as medium quality, and a score less than 50% as low quality. *High quality articles, >70%.

**Table 4.** Critical appraisal skills programme checklist for cohort studies.

Report citation	Groups comparable	Cases and controls matched	Same criteria used for identification of cases and controls	Exposure (physical activity) is measured in a standard, valid and reliable way	Exposure measured in the same way for cases and controls	Confounding factors identified	Strategies used to deal with confounding factors	Outcomes (non-motor symptoms) assessed in a standard, valid and reliable way for cases and controls	Exposure period of interest was long enough to be meaningful	Appropriate statistical analyses used	Total score
Amara et al. (2019)^47^	Y	U	Y	N	Y	Y	Y	Y	Y	Y	80%*

Abbreviations: Y = yes; N = no; U = unclear. Identification of confounding factors was rated ‘Y’ if typical confounders, such as age, gender, Levodopa dosage equivalent, disease duration, and motor symptom severity, were identified and measured. The use of valid and reliable measures was rated ‘Y’ if psychometric properties have been previously evaluated and shown acceptable levels in people with Parkinson's disease. Study quality is defined as follows: a total score greater than 70% as high quality, a score between 50% and 70% as medium quality, and a score less than 50% as low quality. *High quality articles, >70%.

### Articles excluded from meta-analysis

Four articles were not included in the meta-analysis. Alwardat et al*.*^73^ did not contain sufficient information to compute an effect size for statistical pooling. This article reported improvement in anxiety and depression with increases in the weekly physical activity. The methodological heterogeneity of the remaining four articles by Dontje et al.,^76^ Santos et al.,^72^ Leavy et al.,^80^ and Bonde-Jensen et al.^71^ precluded their inclusion in the meta-analysis.

## Discussion

This review showed emerging evidence that people with Parkinson's who are more physically active in their daily lives have better global cognition and less anxiety, apathy, and depression. The prevalence of cognitive impairment and affective disorders is relatively high in people with Parkinson's and contributes to a poor quality of life.^87–90^ While the benefits of physical activity on these symptoms may be independent of each other,^91^ other reports suggest the cognitive benefits of physical activity may be mediated through improvements in depression, anxiety, and apathy.^92–94^ A recent report by Engels et al.^93^ explored interactions among cognition, affective disorders, sleep, fatigue, and pain in people with Parkinson's. Significant positive associations were found among all symptoms, with the strongest association between cognition and affective disorders. Cognitive benefits may also be mediated through less affective symptoms facilitating greater physical activity engagement.^94^ Similarly, improved cognition, such as executive function, may facilitate greater physical activity engagement and therefore the benefits of physical activity on affective symptoms.^95^

Physical activity refers to any bodily movement produced by skeletal muscles that results in energy expenditure, such as walking, work activities, self-care, household activities and sports.^96^ Exercise is a form of physical activity that is planned, structured, repetitive, and performed with the intention of improving or maintaining physical fitness.^96^ Physical activities in free-living conditions refer to leisure activities (e.g. walking, swimming, gardening, golf, dance), non-leisure activities (e.g. transportation, occupational, housework), and planned exercise (e.g. gym, community exercise class) that people do in their everyday life.^27^ The benefits of exercise and physical activity for symptoms experienced by people with Parkinson's may be mediated via a wide range of factors.^97–99^ Among these factors are the increase in endogenous dopamine, serotonin, noradrenaline, and acetylcholine^100–102^ which are depleted in people with Parkinson's who present with non-motor symptoms.^103^ The benefits may also be related to exercise increasing the absorption and transportation of Levodopa across the blood–brain barrier, which enhances endogenous dopamine production.^104,105^

Many people with Parkinson's reduce the amount and intensity of their physical activities, particularly moderate-intensity activity as the disease progresses.^69,70,106^ In contrast, the prevalence and severity of non-motor symptoms increase.^107^ Given the progressive nature of Parkinson's and the known benefits of being physically active, it is important that people with Parkinson's can participate in physical activity recommendations that are sustainable.^22^ Recommendations to increase current free-living leisure, non-leisure, and planned exercise activities is one solution to encourage greater physical activity engagement.^21^

This systematic review suggested that increases in physical activity are associated with changes in some non-motor symptoms; however, the results should be interpreted with caution due to the nature of the observational data, which prevents the assessment of causal relationships. It is also possible that increases in non-motor symptoms are associated with changes in physical activity behaviours. Non-motor symptoms, such as cognition difficulties,^74^ depression,^37–39^ apathy,^108^ fatigue,^38^ excessive daytime sleepiness,^38^ and pain,^40^ are argued to correlate with low physical activity levels. This review did not explore the effect of non-motor symptoms on physical activity behaviour. People with Parkinson's who experience motor impairments, fear of falling, low self-efficacy for exercise, negative perceptions of exercise, access, and resource constraints may further limit their participation in physical activity and contribute to a sedentary lifestyle.^37–41^ There is a need for further research to assess the causal relationship between non-motor symptoms and physical activity in people with Parkinson's with more appropriate designs such as a systematic review of randomised controlled trials.

There were several limitations of this review. No eligible articles included individuals with atypical parkinsonism, so the findings may not be generalisable to this population. However, it is likely that the eligible studies included participants with atypical parkinsonism, but were not identified because they can be indistinguishable from idiopathic Parkinson's due to symptom overlap, particularly in the early stages of the disease.^109,110^ Many articles used retrospective questionnaires, which are inherently subject to recall bias, with participants being more likely to over or underestimate their activity and symptoms.^111–113^ Most investigations assessed few and varied physical activity characteristics (e.g. total weekly physical activity minutes, daily light-, moderate-, and vigorous-intensity activity time, daily steps, and total daily energy expenditure) and used different measurement tools to evaluate non-motor symptoms. This heterogeneity limited the number of articles that could be sensibly pooled in the meta-analyses. While we are confident that our search strategy was comprehensive, having a second reviewer screen titles and abstracts would have ensured no relevant articles were missed. Including non-English language publications and qualitative study designs may have provided this review with a greater breadth and depth of information. In addition, including randomised controlled trials would have provided the review with more detail about exercise dosage (e.g. type, intensity, duration, and frequency).

There is emerging evidence of an association between increased levels of physical activity performed under free-living conditions and better cognitive function and less affective disorder symptoms, especially anxiety, depression, and apathy in people with Parkinson's. There is a need for further research to identify feasible and sustainable physical activity recommendations as such behaviour might slow the rate of functional decline and symptom presentation.^13,14^

**Clinical messages**

- Supporting people with Parkinson's to maintain or increase their usual daily activities affords them a sustainable approach to managing non-motor symptoms
- Participation in physical activities in free-living conditions is related to better cognition and less anxiety, apathy, and depression in people living with Parkinson's disease

## Supplemental Material

sj-docx-1-cre-10.1177_02692155241272967 - Supplemental material for Relationships between physical activities performed under free-living conditions and non-motor symptoms in people with Parkinson's: A systematic review and meta-analysis Supplemental material, sj-docx-1-cre-10.1177_02692155241272967 for Relationships between physical activities performed under free-living conditions and non-motor symptoms in people with Parkinson's: A systematic review and meta-analysis by Amanda Still, Leigh Hale, Sarfaraz Alam, Meg E. Morris and Prasath Jayakaran in Clinical Rehabilitation
