# Supplementary material for: Relationships between physical activities performed under free-living conditions and non-motor symptoms in people with Parkinson's: A systematic review and meta-analysis
Source: Clin Rehabil. 2024 Aug 23;38(11):1534–51. doi: 10.1177/02692155241272967 (PMC11528973; doi:10.1177/02692155241272967)
Supplement: sj-docx-1-cre-10.1177_02692155241272967 - Supplemental material for Relationships between physical activities performed under free-living conditions and non-motor symptoms in people with Parkinson's: A systematic review and meta-analysis [file sj-docx-1-cre-10.1177_02692155241272967.docx]

Supplementary File

JBI Critical Appraisal Checklist for
analytical cross sectional studies

Reviewer______________________________________ Date_______________________________

Author_______________________________________

Year_________ Record Number_________

|  | Yes | No | Unclear | Not applicable |
| --- | --- | --- | --- | --- |
| 1. Were the criteria for inclusion in the sample clearly defined? | □ | □ | □ | □ |
| 1. Were the study subjects and the setting described in detail? | □ | □ | □ | □ |
| 1. Was the exposure measured in a valid and reliable way? | □ | □ | □ | □ |
| 1. Were objective, standard criteria used for measurement of the condition? | □ | □ | □ | □ |
| 1. Were confounding factors identified? | □ | □ | □ | □ |
| 1. Were strategies to deal with confounding factors stated? | □ | □ | □ | □ |
| 1. Were the outcomes measured in a valid and reliable way? | □ | □ | □ | □ |
| 1. Was appropriate statistical analysis used? | □ | □ | □ | □ |

Overall appraisal: Include □ Exclude □ Seek further info □

Comments (Including reason for exclusion)

JBI Critical Appraisal Checklist for
case control studies

Reviewer______________________________________ Date_______________________________

Author_______________________________________

Year_________ Record Number_________

|  | Yes | No | Unclear | Not applicable |
| --- | --- | --- | --- | --- |
| 1. Were the groups comparable other than the presence of disease in cases or the absence of disease in controls? | □ | □ | □ | □ |
| 1. Were cases and controls matched appropriately? | □ | □ | □ | □ |
| 1. Were the same criteria used for identification of cases and controls? | □ | □ | □ | □ |
| 1. Was exposure measured in a standard, valid and reliable way? | □ | □ | □ | □ |
| 1. Was exposure measured in the same way for cases and controls? | □ | □ | □ | □ |
| 1. Were confounding factors identified? | □ | □ | □ | □ |
| 1. Were strategies to deal with confounding factors stated? | □ | □ | □ | □ |
| 1. Were outcomes assessed in a standard, valid and reliable way for cases and controls? | □ | □ | □ | □ |
| 1. Was the exposure period of interest long enough to be meaningful? | □ | □ | □ | □ |
| 1. Was appropriate statistical analysis used? | □ | □ | □ | □ |

Overall appraisal: Include □ Exclude □ Seek further info □
Comments (Including reason for exclusion)

________________________________________________________________________________________________________

**Figure S1.** JBI critical appraisal checklist for analytical cross-sectional and case control studies.

**Critical Appraisal Skills Programme Checklist for Cohort Studies**

| **Section A: Are the results of the study valid?** | | |
| --- | --- | --- |
| 1. Did the study address a clearly focused issue? | □ Yes  □ No  □ Can’t Tell | HINT: A question can be ‘focused’ in terms of   - the population studied - the risk factors studied - is it clear whether the study tried to detect a beneficial or harmful effect   the outcomes considered |
| 1. Was the cohort recruited in an acceptable way? | □ Yes  □ No  □ Can’t Tell | HINT: Look for selection bias which might compromise the generalisability of the findings:   - was the cohort representative of a defined population - was there something special about the cohort - was everybody included who should have been |
| 1. Was the exposure accurately measured to minimise bias? | □ Yes  □ No  □ Can’t Tell | HINT: Look for measurement or classification bias:   - did they use subjective or objective measurements - do the measurements truly reflect what you want them to (have they been validated) - were all the subjects classified into exposure groups using the same procedure |
| 1. Was the outcome accurately measured to minimise bias? | □ Yes  □ No  □ Can’t Tell | HINT: Look for measurement or classification bias:   - did they use subjective or objective measurements - do the measurements truly reflect what you want them to (have they been validated) - has a reliable system been established for detecting all the cases (for measuring disease occurrence) - were the measurement methods similar in the different groups - were the subjects and/or the outcome assessor blinded to exposure (does this matter) |
| 1. Have the authors identified all important confounding factors? | □ Yes  □ No  □ Can’t Tell | HINT:   - list the ones you think might be important, and ones the author missed |
| 1. Have they taken account of the confounding factors in the design and/or analysis? | □ Yes  □ No  □ Can’t Tell | HINT:  look for restriction in design, and techniques e.g. modelling, stratified-, regression-, or sensitivity analysis to correct, control or adjust for confounding factors |
| 1. Was the follow up of subjects complete enough? | □ Yes  □ No  □ Can’t Tell | HINT: Consider   - the good or bad effects should have had long enough to reveal themselves - the persons that are lost to follow-up may have different outcomes than those available for assessment - in an open or dynamic cohort, was there anything special about the outcome of the people leaving, or the exposure of the people entering the cohort |
| 1. Was the follow up of subjects long enough? | □ Yes  □ No  □ Can’t Tell |  |
| **Section B: What are the results?** | | |
| 1. What are the results of this study? |  | HINT: Consider   - what are the bottom line results have they reported the rate or the proportion between the exposed/unexposed, the ratio/rate difference how strong is the association between exposure and outcome (RR) - what is the absolute risk reduction (ARR) |
| 1. How precise are the results? |  | HINT: look for the range of the confidence intervals, if given |
| 1. Do you believe the results? | □ Yes  □ No  □ Can’t Tell | HINT: Consider   - big effect is hard to ignore - can it be due to bias, chance or confounding - are the design and methods of this study sufficiently flawed to make the results unreliable - Bradford Hills criteria (e.g. time sequence, dose-response gradient, biological plausibility, consistency) |
| **Section C: Will the results help locally?** | | |
| 1. Can the results be applied to the local population? | □ Yes  □ No  □ Can’t Tell | HINT: Consider   - whether a cohort study was the appropriate method to answer this question the subjects covered in this study could be sufficiently different from your population to cause concern - your local setting is likely to differ much from that of the study you can quantify the local benefits and harms |
| 1. Do the results of this study fit with other available evidence? | □ Yes  □ No  □ Can’t Tell |  |

**Figure S2.** Critical Appraisal Skills Programme Checklist for Cohort Studies.

1. $\beta= \frac{SD (X)}{SD (Y)}b$
2. $SE(\beta\mathrm{or}b)=\left( upper limit CI-lower limit CI \right)/3.92$
3. $\mathrm{SE}\left( \beta\right)= \frac{\mathrm{SD}\left( X \right)}{\mathrm{SD}\left( Y \right)}\mathrm{SE}\left( b \right)$
4. $\mathrm{SD}\left( Y \right)= \frac{(maximum-minimum)}{6}$
5. $\mathrm{SD}\left( Y \right)=\frac{(upper quartile-lower quartile)}{1.349}$
6. $95\%CI \left( \beta\right)= \beta\frac{+}{-} t\left( 0.975\left( n-1 \right) \right)SE(\beta)$
7. $\mathrm{SE}\left( \beta\right)= \frac{1- r^{2}}{\sqrt{n-1}}$
8. *r_s_ ≈ r* $=$ $\beta$ *(from univariate model)*

**Figure S3.** Formulas used to transform correlation coefficients and unstandardized regression coefficients into standardized regression coefficients with 95% confidence interval.

**Figure S4.** Forest plots with statistically non-significant pooled estimates from articles estimating the relationship between physical activity categories and non-motor symptoms in Parkinson’s disease.

**Table S1.** Search strategy domains with keywords

| Domain | 1 | 2 | 3 | 4 | 5 | 6 |
| --- | --- | --- | --- | --- | --- | --- |
|  | Parkinson’s | Non-motor symptoms | Physical Activity | Relationship | Study design | Study design |
| Boolean Operators | AND | AND | AND | AND | AND | NOT |
| Key words | Parkinson* OR "Parkinson* disease" OR "idiopathic parkinson* disease" OR "primary parkinson*" OR "paralysis agitans" OR "shaking palsy" OR "parkinson* disorder*" OR "lewy body parkinson* disease" OR "progressive supranuclear palsy" OR "corticobasal degeneration" OR "corticobasal syndrome" OR "multiple systems atrophy" OR "Shy-Drager syndrome" OR "olivopontocerebellar atrophy" OR "striatonigral degeneration" OR "dementia with lewy bodies" OR "parkinson* plus syndrome*" OR "atypical parkinson*" OR synucleinopath* OR tauopath* | "Non motor symptom*" OR "non motor sign*" OR "non motor feature*" OR "non-motor symptom scale" OR sleep* OR "sleep disorder*" OR "dyssomnia*" OR "parasomnia*" OR "insomnia*" OR "hypersomnia*" OR "sleep fragmentation" OR "daytime sleepiness" OR "sleep apnea" OR "sleep apnoea" OR "sleep disordered breathing" OR "rapid eye movement behaviour disorder" OR "REM behaviour disorder" OR "sleep quality" OR "circadian rhythm sleep disorder*" OR "periodic limb movement*" OR "restless leg*" OR pain OR "pain perception*" OR fatigu* OR "muscle fatigue" OR "mental fatigue" OR "fatigue syndrome" OR Cogniti* OR "cognitive function*" OR "cognitive disorder*" OR "cognitive dysfunction*" OR "dementia" OR "mood disorder*" OR "mood disturbance*" OR "affective disorder*" OR anxiet* OR depress* OR "depressive disorder" OR anhedonia* OR apathy OR "quality of life" | "Physical activit*" OR exercise OR "exercise behaviour" OR "exercise behavior" OR "sedentary behaviour" OR "sedentary behavior" OR "leisure activit*" OR "recreation" OR walk* OR "activity level*" OR "daily activit*" OR "activities of daily living" OR "habitual physical activit*" | Relationship* OR correlat* OR associat* OR interact* OR determinant* OR predict* | "Observational study" OR "cross-sectional study" OR "longitudinal study" | "Randomised controlled trial" OR "randomized controlled trial" OR "clinical trial" OR "controlled clinical trial" OR "systematic review" OR review |

**Table S2.** Filters and limits used in each database

| **Filters & Limits** | **Databases** | | | | | |
| --- | --- | --- | --- | --- | --- | --- |
|  | Scopus | Web of Science | Ovid PsycINFO | PubMed | CINHAL | ProQuest  (Health and Medicine) |
|  | PUBYEAR  >  1999  SRCTYPE ,  "j"  DOCTYPE ,  "ar"  LANGUAGE ,  "English"  EXACTKEYWORD ,  "Humans" | Articles,  English  Publication year: 2000-2021 | Human,  Peer-reviewed journal,  journal article,  English,  last 21 years | Journal Article, Humans, English, from 2000 - 2021*.* | Published Date: 2000-2021; Human; Journal Subset: Peer Reviewed; Publication Type: Journal Article; Language: English | Date from 2000 to 2021, scholarly journals, articles, English |

**Table S3.** Data extraction template for description of included studies.

| Study Citation/Design | Recruitment country | PD sample size and gender  (*n*, % Male) | Diagnosis | Setting | Age, years (Mean, [SD]) ^a^ | Disease duration, years  (Mean, [SD]) ^a^ | Disease stage, Original H&Y Scale (*n,* [%]) ^a^ | MDS-UPDRS motor examination score (Mean, [SD]) ^a^ | LEDD, mg (Mean, [SD]) ^a^ |
| --- | --- | --- | --- | --- | --- | --- | --- | --- | --- |
|  | - | - | - | - | - | - | - | - | - |

Abbreviations: *n*, number; SD, Standard deviation; PD, Parkinson’s disease; H&Y, Hoehn and Yahr Scale; MDS-UPDRS, International Parkinson and Movement Disorder Society – Unified Parkinsons Disease Rating Scale; LEDD, Levodopa Equivalent Daily Dosage.
^a^ Except where indicated

**Table S4.** Data extraction template for observed standardized regression coefficient $\beta$ (effect size) with standard error SE ($\beta$) and 95% confidence interval, from studies estimating the relationship between physical activity and non-motor symptoms in Parkinson’s.

| Citation | Reported effect size $(\beta, b, r_{s})$ | β | SE(β) | Lower Limit of 95% CI | Upper Limit of 95% CI | Sample Size | Covariates | Physical Activity  Measure (X)  (SD) | NMS  Measure (Y)  (SD) |
| --- | --- | --- | --- | --- | --- | --- | --- | --- | --- |
|  |  |  |  |  |  |  |  |  |  |

Abbreviations: $\beta,$Standardized beta coefficient; $b,$ unstandardized beta coefficient; $r_{s},$Spearman’s correlation coefficient; SE, Standard Error; CI, Confidence Interval; SD, Standard Deviation.

**Table S5.** Observed standardized regression coefficient $\beta$ with standard error SE ($\beta$) and 95% confidence interval, sample size and weight in pooled analysis from studies estimating the relationship between physical activity and non-motor symptoms in Parkinson’s.

| **Citation** | **Reported effect size** | **β** | **SE(β)** ^c^ | **Lower Limit of 95% CI** | **Upper Limit of 95% CI** | **Sample Size** | **Covariates** | **Physical Activity  Measure (X)** | **NMS  Measure (Y)** |
| --- | --- | --- | --- | --- | --- | --- | --- | --- | --- |
| **COGNITION** |  |  |  |  |  |  |  |  |  |
| **Weekly TPA** |  |  |  |  |  |  |  |  |  |
| Duvdevani et al. (2024) | *r* | 0.255* | 0.100 | 0.0481 | 0.4409 | 88 | None | IPAQ (MET-min/wk)  (SD = 2127.04) | PDQ-39 Cog (0-100%) ^#^  (SD = 21.23) |
| Ough et al. (2014) | $r_{s}$ | 0.133* | 0.021 | 0.092 ^c^ | 0.170 ^c^ | 2252 | None | PA (min/week)  Baseline  (SD = 427.07) ^I^ | PDQ-39 Cog (0-100%) ^#^  Baseline  (SD = 22.1) ^I^ |
|  | $b$ | -0.000 | Insufficient data to compute | | |  | Age, sex, disease duration, H&Y, cognition |  |  |
|  |  | 0.000 | Insufficient data to compute | | |  |  |  | $\Delta$ PDQ-39 Cog (0-100%) ^#^  1Y Follow-up  (SD = 22.1) ^I^ |
| Shih et al. (2019) | $r_{s}$ | 0.227* | 0.072 | 0.082 | 0.350 | 174 | None | PASE (0-793)  (SD = 90.12) | MoCA (0-30)  (SD = 3.01) |
| Ng et al. (2021) | $\beta$ | 0.040 | 0.281 | -0.510 | 0.590 | 121 | None | PASE (0 – 793)  Baseline  (SD = 89.51) | MoCA (0-30)  1Y follow-up  (SD = 3.5) |
|  |  | -0.037 | 0.111 | -0.255 | 0.181 |  | Age, gender, ethnicity, years of education, MDS-UPDRS Part III, LEDD, motor subtypes |  |  |
| Amara et al. (2019) | $b$ | -0.004 ^c^ | 0.055 | -0.112 ^c^ | 0.104 ^c^ | 370 | Age, gender, disease duration | PASE (0 – 793)  Baseline  (SD = 100.7) | MoCA (0-30)  1Y follow-up (SD = 3.2) |
|  |  | 0.114 *^c^ | 0.047 | 0.02158 | 0.2064 ^c^ | 370 |  |  | MoCA (0-30)  2Y follow-up (SD = 3.2) |
|  |  | -0.069 ^c^ | 0.071 | -0.209 ^c^ | 0.071 ^c^ | 301 | Age, gender, disease duration, MDS-UPDRS Part III score |  | MoCA (0-30)  1Y follow-up (SD = 3.2) |
|  |  | 0.079 ^c^ | 0.059 | -0.0371 ^c^ | 0.1951 ^c^ | 301 |  |  | MoCA (0-30)  2Y follow-up (SD = 3.2) |

**Table S5.** (Continued)

| **Citation** | **Reported effect size** | **β** | **SE(β)** ^c^ | **Lower Limit of 95% CI** | **Upper Limit of 95% CI** | **Sample Size** | **Covariates** | **Physical Activity  Measure (X)** | **NMS  Measure (Y)** |
| --- | --- | --- | --- | --- | --- | --- | --- | --- | --- |
| **COGNITION** |  |  |  |  |  |  |  |  |  |
| **Daily MVPA** |  |  |  |  |  |  |  |  |  |
| Ellingson et al. (2019) | $r_{s}$ | 0.129 | 0.142 | -0.150 ^c^ | 0.390 ^c^ | 52 | None | IPAQ: Past 7 days (min/day)  (SD = 61.01) | PDQ-39 Cognition  (0-100%) ^#^  (SD = 22.1) |
|  |  | 0.170 | 0.144 | -0.108 ^c^ | 0.423 ^c^ | 52 |  | Accelerometery (min/day)  (SD = 39.88) |  |
| Cerff et al. (2017) | $r_{s}$ | 0.190 | 0.141 | -0.390 | 0.150 | 48 | None | Accelerometery (% of 24 hrs)  (Moderate)  (SD =0.018) ^c^ | MMSE (0-30)  (SD = 1.7) ^I^ |
|  |  | 0.150 | 0.143 | -0.140 | 0.420 | 48 |  | Accelerometery (% of 24 hrs) (Vigorous)  (SD = 0.0056) ^c^ |  |
| Donahue et al. (2022) | $r_{s}$ | 0.295* | 0.094 | 0.101 ^c^ | 0.468 ^c^ | 96 | None | Accelerometery (min/day) (SD = 16.7) | MoCA (0-30)  (SD = 3.2) ^I^ |
|  |  | 0.398* | 0.086 | 0.215 ^c^ | 0.554 ^c^ |  |  |  | Global cognition neuropsychological battery  (SD = NR) |
|  | $b$ | 0.209* | 0.097 | 0.016 ^c^ | 0.402 ^c^ |  | Age, sex, MDS-UPDRS motor score, and self- reported exercise duration |  | MoCA (0-30)  (SD = 3.2) ^I^ |
|  |  | 0.015 ($b$) | 0.005($b$) | 0.005 ($b$) | 0.025 ($b$) |  |  |  | Global cognition neuropsychological battery  (SD = NR) |
| Loprinzi et al. (2018) | $\beta$ | 0.090* | 0.047 | 0.004 | 0.190 | 23 | Age, Gender, H&Y | Accelerometery (min/day)  (SD = 39.88) ^I^ | MoCA (0-30)  (SD = 3.2) ^I^ |

**Table S5.** (Continued)

| **Citation** | **Reported effect size** | **β** | **SE(β)** ^c^ | **Lower Limit of 95% CI** | **Upper Limit of 95% CI** | **Sample Size** | **Covariates** | **Physical Activity  Measure (X)** | **NMS  Measure (Y)** |
| --- | --- | --- | --- | --- | --- | --- | --- | --- | --- |
| **COGNITION** |  |  |  |  |  |  |  |  |  |
| **Daily LPA** |  |  |  |  |  |  |  |  |  |
| Cerff et al. (2017) | $r_{s}$ | 0.110 | 0.144 | -0.160 ^c^ | 0.380 ^c^ | 48 | None | Accelerometery (% of 24hrs)  (SD = 0.052) ^c^ | MMSE (0-30)  (SD = 1.7) ^I^ |
| Donahue et al. (2022) | $r_{s}$ | 0.275* | 0.095 | 0.079 ^c^ | 0.451 ^c^ | 96 | None | Accelerometery (min/day) (SD = 97.01) | MoCA (0-30)  (SD = 3.2) ^I^ |
|  |  | 0.232* | 0.097 | 0.033 ^c^ | 0.413 ^c^ |  |  |  | Global cognition neuropsychological battery  (SD = NR) |
|  | $b$ | 0.091 | 0.085 | -0.078 | 0.260 |  | Age, sex, MDS-UPDRS motor score, and self- reported exercise duration |  | MoCA (0-30)  (SD = 3.2) ^I^ |
|  |  | 0.000($b$) | 0.001($b$) | -0.002($b$) | 0.002 ($b$) |  |  |  | Global cognition neuropsychological battery  (SD = NR) |
| **Daily Steps** |  |  |  |  |  |  |  |  |  |
| Cerff et al. (2017) | $r_{s}$ | 0.080 | 0.145 | -0.220 | 0.360 | 48 | None | Accelerometery  (SD = 2,081) ^c^ | MMSE (0-30)  (SD = 1.7) ^I^ |
| Ellingson et al. (2019) | $r_{s}$ | 0.231 | 0.148 | -0.045 ^c^ | 0.474 ^c^ | 52 | None | Accelerometery  (SD = 3131.7) | PDQ-39 Cognition (0-100%) ^#^  (SD = 22.1) |
| **Daily EE** |  |  |  |  |  |  |  |  |  |
| Dontje et al. (2013) | $r_{s}$ | 0.120* | 0.046 | 0.030 ^c^ | 0.208 ^c^ | 467 | None | Accelerometery (Kcal/day)  (SD = 201.4) ^c^ | MMSE (0-30)  (SD = 1.7) |

**Table S5.** (Continued)

| **Citation** | **Reported effect size** | **β** | **SE(β)** ^c^ | **Lower Limit of 95% CI** | **Upper Limit of 95% CI** | **Sample Size** | **Covariates** | **Physical Activity  Measure (X)** | **NMS  Measure (Y)** |
| --- | --- | --- | --- | --- | --- | --- | --- | --- | --- |
| **COGNITION** |  |  |  |  |  |  |  |  |  |
| **Monthly PA days** |  |  |  |  |  |  |  |  |  |
| Santos et al. (2018) | *β* | 0.010 | 0.099 | -0.180 | 0.210 | 130 | Age, gender, GDS, education level, severity of MPS, global health score | PA (days/month) *Baseline*  (SD = 10.96) | RBANS (40-160) *Baseline*  (SD = 11.85) |
|  |  | 0.350* | 0.110 | 0.130 | 0.560 | 130 |  | PA (days/month)  *1Y Follow-up*  (SD = 10.55) | RBANS (40-160)  *1Y Follow-up*  (SD = NR) |
|  |  | 0.240* | 0.066 | 0.110 | 0.370 | 130 |  | $\Delta$PA (days/month)  *1Y Follow-up*  (SD = 12.79) | $\Delta$RBANS (40-160)  *1Y Follow-up*  (SD = 7.53) |
| **AFFECT** |  |  |  |  |  |  |  |  |  |
| **Weekly TPA** |  |  |  |  |  |  |  |  |  |
| Amara et al. (2019) | $b$ | 0.093* | 0.029 | 0.150 ^c^ | 0.036 ^c^ | 370 | Age, gender, disease duration | PASE (0 – 793)  (SD = 100.7) | STAI (20-80) ^#^  (SD = 18.5) |
|  |  | 0.104* | 0.035 | 0.173 ^c^ | 0.035 ^c^ |  |  |  | GDS (0-15) ^#^  (SD =2.9) |
|  |  | 0.104* | 0.035 | 0.173 ^c^ | 0.035 ^c^ | 301 | Age, gender, disease duration, MDS-UPDRS Part III score |  | GDS (0-15) ^#^  (SD =2.9) |
|  |  | 0.114* | 0.035 | 0.183 ^c^ | 0.045 ^c^ |  |  |  | STAI (20-80) ^#^  (SD = 18.5) |

**Table S5.** (Continued)

| **Citation** | **Reported effect size** | **β** | **SE(β)** ^c^ | **Lower Limit of 95% CI** | **Upper Limit of 95% CI** | **Sample Size** | **Covariates** | **Physical Activity  Measure (X)** | **NMS  Measure (Y)** |
| --- | --- | --- | --- | --- | --- | --- | --- | --- | --- |
| **AFFECT** |  |  |  |  |  |  |  |  |  |
| **Weekly TPA** |  |  |  |  |  |  |  |  |  |
| Duvdevani et al. (2024) | *r* | 0.364* | 0.093 | 0.167 | 0.533 | 88 | None | IPAQ (MET-min/wk)  (SD = 2127.04) | PDQ-39 Emotion  (0-100%) ^#^  (SD = 24.84) |
| Ng et al. (2021) | $\beta$ | 0.240 | 0.648 | -1.030 | 1.510 | 121 | None | PASE (0 – 793)  (SD = 89.51) | AS (0-42) ^#^  (SD = 6.27) |
|  |  | -0.050 | 0.235 | -0.510 | 0.410 |  |  |  | HADS-D (0-21) ^#^  (SD= 3.16) |
|  |  | -0.600* | 0.204 | 0.200 | 1.000 |  |  |  | HADS-A (0-21) ^#^  (SD= 3.18) |
|  |  | 0.330* | 0.138 | 0.060 | 0.600 |  | Age, gender, ethnicity, education years, MDS-UPDRS Part III, LEDD, motor subtypes, AS, HADS, FSS, ESS, PSQI |  | AS (0-42) ^#^  (SD = 6.27) |
|  |  | -0.120 | 0.143 | -0.340 | 0.220 |  |  |  | HADS-D (0-21) ^#^  (SD= 3.16) |
|  |  | 0.270* | 0.130 | 0.010 | 0.520 |  |  |  | HADS-A (0-21) ^#^  (SD= 3.18) |
| Ough et al. (2014) | $r_{s}$ | 0.151* | 0.021 | 0.110 ^c^ | 0.191 ^c^ | 2252 | None | PA (min/week)  (SD = 427.07) ^I^ | PDQ-39 Emotion^#^  (SD = 15.5) ^I^ |
|  | $b$ | -0.827 | *Insufficient information available to compute* | | Age, sex, disease duration, H&Y, cognition |  |  |  |  |
|  |  | 1.929* |  |  |  |  |  |  | $\Delta$PDQ-39 Emotion ^#^  *1-Y Follow-up*  (SD = 15.5) ^I^ |
| Alwardat et al. (2019) | $b$ | NR* | 0.064 ($b$) | 0.330 ($b$) | 0.580 ($b$) | 128 | Age, sex, UPDRS III score, and disease duration, LEDD | IPAQ-SF (MET-min/wk)  (SD = 2197.53) | HAM-A (0-56) ^#^  (SD = 8.71) |
|  |  | NR* | 0.079 ($b$) | 0.310 ($b$) | 0.620 ($b$) |  |  |  | HAM-D (0-56) ^#^  (SD = 8.46) |

**Table S5.** (Continued)

| **Citation** | **Reported effect size** | **β** | **SE(β)** ^c^ | **Lower Limit of 95% CI** | **Upper Limit of 95% CI** | **Sample Size** | **Covariates** | **Physical Activity  Measure (X)** | **NMS  Measure (Y)** |
| --- | --- | --- | --- | --- | --- | --- | --- | --- | --- |
| **AFFECT** |  |  |  |  |  |  |  |  |  |
| **Daily MVPA** |  |  |  |  |  |  |  |  |  |
| Ellingson et al. (2019) | $r_{s}$ | 0.044 | 0.140 | -0.232 ^c^ | 0.313 ^c^ | 52 | None | Accelerometery  (min/day)  (SD = 39.88) | PDQ-39 Emotion ^#^  (SD = 15.5) |
|  |  | -0.041 | 0.140 | -0.310 ^c^ | 0.235 ^c^ |  |  | IPAQ: Past 7 days (min/day)  (SD = 61.01) |  |
| **Daily steps** |  |  |  |  |  |  |  |  |  |
| Ellingson et al. (2019) | $r_{s}$ | 0.090 | 0.139 | -0.188 ^c^ | 0.345 ^c^ | 52 | None | Accelerometery  (SD = 3131.7) | PDQ-39 Emotion ^#^  (SD = 15.5) |
| Leavy et al. (2021) | $r$ | 0.195 | 0.103 | -0.018 | 0.384 | 89 | None | Accelerometery  (SD = 3180) | HADS-D ^#^  (SD = 2.97) |
| **Daily EE** |  |  |  |  |  |  |  |  |  |
| Dontje et al. (2012) | $r_{s}$ | -0.080 | 0.046 | -0.170 ^c^ | 0.011 ^c^ | 467 | None | Accelerometery  (kcal/day)  (SD = 201.41) | HADS-A (0-21) ^#^  (SD = 3.4) |
|  |  | 0.010 | 0.046 | -0.081 ^c^ | 0.101 ^c^ |  |  |  | HADS-D (0-21) ^#^  (SD = 3.6) |

**Table S5.** (Continued)

| **Citation** | **Reported effect size** | **β** | **SE(β)** ^c^ | **Lower Limit of 95% CI** | **Upper Limit of 95% CI** | **Sample Size** | **Covariates** | **Physical Activity  Measure (X)** | **NMS  Measure (Y)** |
| --- | --- | --- | --- | --- | --- | --- | --- | --- | --- |
| **SLEEP** |  |  |  |  |  |  |  |  |  |
| **Weekly TPA** |  |  |  |  |  |  |  |  |  |
| Ng et al. (2021) | $\beta$ | -0.010 | 0.133 | -0.270 | 0.250 | 121 | Age, gender, ethnicity, education years, MDS-UPDRS Part III, LEDD, motor subtypes, AS, HADS, FSS, ESS, PSQI | PASE *Baseline*  (SD = 89.51) | PSQI^#^ (0-21) *1Y Follow-up*  (SD= 3.3) |
|  |  | -0.06 | 0.140 | -0.34 | 0.21 |  |  |  | ESS ^#^ (0-24) *1Y Follow-up*  (SD = 4.5) |
|  |  | 0.060 | 0.288 | -0.510 | 0.620 |  | None |  | PSQI^#^ (0-21) *1Y Follow-up*  (SD= 3.3) |
|  |  | 0.060 | 0.436 | -0.800 | 0.91 |  |  |  | ESS ^#^ (0-24) *1Y Follow-up*  (SD = 4.5) |
| Amara et al. (2019) | $b$ | 0.027 ^c^ | 0.024 | -0.020 ^c^ | 0.074 ^c^ | 370 | Age, gender, disease duration | PASE *Baseline*  (SD = 100.7) | REMSBDQ ^#^ (0-13) *2Y Follow-up*  (SD = *NR*) |
|  |  | 0.000 ^c^ | 0.037 | -0.073 ^c^ | 0.073 ^c^ |  |  |  | ESS ^#^ (0-24) *2Y Follow-up*  (SD = 4.1) |
|  |  | 0.013 ^c^ | 0.043 | -0.072 ^c^ | 0.098 ^c^ |  |  |  | MDS-UPDRS item Sleep Score ^#^ (0-8) *2Y Follow-up*  (SD = 1.6) |
|  |  | 0.003 | 0.027 | -0.050 | 0.056 | 301 | Age, gender, disease duration, MDS-UPDRS part III | PASE *Baseline*  (SD = 100.7) | REMSBDQ ^#^ (0-13) *2Y Follow-up*  (SD = *NR*) |
|  |  | 0.020 ^c^ | 0.037 | -0.053 ^c^ | 0.093 ^c^ |  |  |  | ESS ^#^ (0-24) *2Y Follow-up*  (SD = 4.1) |
|  |  | 0.071* ^c^ | 0.034 | 0.004 ^c^ | 0.138 ^c^ |  |  |  | MDS-UPDRS Sleep Score^#^ (0-8) *1Y Follow-up*  (SD = 1.6) |
|  |  | 0.164* ^c^ | 0.071 | 0.024 ^c^ | 0.304 ^c^ |  |  |  | MDS-UPDRS Sleep Score^#^  (0-8) *2Y Follow-up*  (SD = 1.6) |

**Table S5.** (Continued)

| **Citation** | **Reported effect size** | **β** | **SE(β)** ^c^ | **Lower Limit of 95% CI** | **Upper Limit of 95% CI** | **Sample Size** | **Covariates** | **Physical Activity  Measure (X)** | **NMS  Measure (Y)** |
| --- | --- | --- | --- | --- | --- | --- | --- | --- | --- |
| **PAIN** |  |  |  |  |  |  |  |  |  |
| **Weekly TPA** |  |  |  |  |  |  |  |  |  |
| Ough et al. (2014) | $r_{s}$ | 0.118* | 0.021 | 0.077 ^c^ | 0.159 ^c^ | 2252 | None | Physical Activity (min/week)  (SD = 427.07) ^I^ | PDQ-39 Discomfort ^#^  (0-100) (SD = 18.53) ^I^ |
|  | $b$ | 0.230 | *Insufficient information available to compute* | | Age, sex, disease duration, H&Y, cognition |  | PDQ-39 Discomfort ^#^  (0-100) (SD = 18.53) ^I^ |  |  |
|  |  | -0.230 |  |  |  |  | $\Delta$PDQ-39 Discomfort ^#^  (0-100) *1Y Follow-up*  (SD = 18.53) ^I^ |  |  |
| Nguy et al. (2020) | $b$ | -0.396* ^c^ | 0.134 | -0.665 ^c^ | -0.127 ^c^ | 52 | None | IPEQ (h/week)  (SD = 15.7) | KPPS_total score ^#^  (0 – 168) (SD = 12.3) |
|  |  | -0.262* ^c^ | 0.156 | -0.575 ^c^ | 0.051 ^c^ |  |  |  | BPI_severity subscore ^#^  (0-10) (SD = 1.8) |
|  | $\beta$ | -0.070 | 0.134 | -0.340 ^c^ | 0.200 ^c^ |  | Disease duration, HADS-A, PSQI, CSI |  |  |
|  |  | -0.25* | 0.107 | -0.465 ^c^ | -0.035 ^c^ |  |  |  | KPPS_total score ^#^  (0 – 168) (SD = 12.3) |
| **Daily MVPA** |  |  |  |  |  |  |  |  |  |
| Ellingson et al. (2019) | $r_{s}$ | -0.021 | 0.140 | -0.29 ^c^ | 0.25 ^c^ | 52 | None | Accelerometery  (SD = 39.88) | PDQ-39 Discomfort ^#^  (0-100) (SD = 18.53) |
|  |  | 0.116 | 0.138 | -0.16 ^c^ | 0.38 ^c^ |  |  | IPAQ: Past 7-days (min/day) (SD = 61.01) |  |
| Nguy et al. (2020) | $b$ | 0.102 ^c^ | 0.146 | -0.191 ^c^ | 0.395 ^c^ | 52 | None | Accelerometery  (% of 24hrs) (SD = 2.8) | KPPS_total score ^#^  (0 – 168) (SD = 12.3) |
|  |  | 0.171 ^c^ | 0.147 | -0.124 ^c^ | 0.466 ^c^ |  |  |  | BPI_severity subscore ^#^  (0-10) (SD = 1.8) |

**Table S5.** (Continued)

| **Citation** | **Reported effect size** | **β** | **SE(β)** ^c^ | **Lower Limit of 95% CI** | **Upper Limit of 95% CI** | **Sample Size** | **Covariates** | **Physical Activity  Measure (X)** | **NMS  Measure (Y)** |
| --- | --- | --- | --- | --- | --- | --- | --- | --- | --- |
| **PAIN** |  |  |  |  |  |  |  |  |  |
| **Daily LPA** |  |  |  |  |  |  |  |  |  |
| Nguy et al. (2020) | $b$ | -0.325* ^c^ | 0.137 | -0.600 ^c^ | -0.050 ^c^ | 52 | None | Accelerometery  (% of 24hrs) (SD = 10.8) | KPPS_total score ^#^  (0 – 168) (SD = 12.3) |
|  |  | -0.240 ^c^ | 0.153 | -0.547 ^c^ | 0.067 ^c^ |  |  |  | BPI_severity subscore ^#^  (0-10) (SD = 1.8) |
| **Daily Steps** |  |  |  |  |  |  |  |  |  |
| Ellingson et al. (2019) | $r_{s}$ | 0.073 | 0.139 | -0.2 ^c^ | 0.34 ^c^ | 52 | None | Accelerometery  (SD = 3131.7) | PDQ-39 Discomfort ^#^  (0-100) (SD = 18.53) |
| Nguy et al. (2020) | $b$ | -0.220 ^c^ | 0.169 | -0.559 ^c^ | 0.119 ^c^ | 52 | None | Accelerometery  (SD = 2708.5) | KPPS_total score ^#^  (0 – 168) (SD = 12.3) |
|  |  | -0.060 ^c^ | 0.154 | -0.369 ^c^ | 0.249 ^c^ |  |  |  | BPI_severity subscore ^#^  (0-10) (SD = 1.8) |
| **FATIGUE** |  |  |  |  |  |  |  |  |  |
| **Weekly TPA** |  |  |  |  |  |  |  |  |  |
| Amara et al. (2019) | $b$ | 0.077 ^c^ | 0.057 | -0.035 ^c^ | 0.189 ^c^ | 370 | Age, gender, disease duration | PASE  *Baseline*  (SD = 100.7) | MDS-UPDRS Item 1.13 score ^#^ (0-4)  *2Y-Follow-up*  (SD = 1.3) ^I1,2^ |
|  |  | 0.062 ^c^ | 0.117 | -0.168 ^c^ | 0.292 ^c^ | 301 | Age, gender, disease duration, MDS-UPDRS part III score |  |  |
| Ng et al. (2021) | $\beta$ | 0.000 | 0.135 | -0.270 | 0.260 | 121 | None | PASE  *Baseline*  (SD = 89.51) | FSS ^#^ (9-63)  *1Y Follow-up*  (SD = 1.49) |
|  |  | 0.030 | 0.115 | -0.190 | 0.260 |  | Age, gender, ethnicity, education years, MDS-UPDRS Part III, LEDD, motor subtypes, AS, HADS, ESS, PSQI |  |  |

**Table S5.** (Continued)

Abbreviations: $\beta,$Standardized beta coefficient; $b,$ unstandardized beta coefficient; $r_{s},$Spearman’s correlation coefficient; $r$, Pearson’s correlation coefficient; SE, Standard Error; CI, Confidence Interval; SD, Standard Deviation. IPAQ, International Physical Activity Questionnaire; PDQ-39, Parkinson’s Disease Questionnaire-39; min/day, minutes per day; MMSE, Mini-Mental State Examination; MoCA, Montreal Cognitive Assessment; NR, Not Reported; MDS-UPDRS Part III, International Parkinson and Movement Disorder Society – Unified Parkinsons Disease Rating Scale, Motor Examination; H&Y, Hoehn and Yahr Scale; GDS, Geriatric Depression Scale; PA, Physical Activity; Kcal/day, Kilocalories per day; RBANS, Repeatable Battery for Assessment of Neuropsychological Status; Y, Year; $\Delta,$ Change; PASE, Physical Activity Scale for Elderly; STAI, State-Trait Anxiety Inventory; AS, Apathy Scale; LEDD, Levodopa Equivalent Daily Dosage; HADS-A, Hospital Anxiety and Depression Scale – Anxiety subscale; HADS-D, Hospital Anxiety and Depression Scale – Depression subscale; FSS, Fatigue Severity Scale; ESS, Epworth sleepiness scale; PSQI, Pittsburgh Sleep Quality Index; HAM-A, Hamilton Anxiety Rating Scale; HAM-D, Hamilton Depression Rating Scale; REMSBDQ, REM Sleep Behaviour Disorder Questionnaire; IPEQ, Incidental and Planned Exercise Questionnaire; h/week, hours per week; CSI, Central Sensitization Inventory; KPPS, King’s PD Pain Scale; BPI, Brief Pain Inventory; MET-min/wk, Metabolic Equivalent minutes per week. ^c^ Computed estimate; *Statistically significant (*p* < 0.05); ^#^ Where higher scores indicate worse symptoms, the direction of the coefficient and its corresponding 95% CI were reversed; ^I^ Imputed data from studies included in review unless otherwise cited; Green data boxes indicate studies included in meta-analyses.

**References for Table S5:**

1. Eghlidos Z, Rahimian Z, Vadiee G, Jahangiri S. Effects of subthalamic deep brain stimulation on non‐motor symptoms of Parkinson's disease: a meta‐analysis. *Acta Neurol Scand*. 2022;146(2):115-125.
2. Chou KL, Taylor JL, Patil PG**.** The MDS−UPDRS tracks motor and non-motor improvement due to subthalamic nucleus deep brain stimulation in Parkinson disease. *Parkinsonism & Related Disorders*. 2013;19(11):966-9.
